# Knowledge and attitudes of pregnant women about endocrine disruptors

**DOI:** 10.1590/1806-9282.20250803

**Published:** 2025-10-27

**Authors:** Sevilay Aydin Çelik, Beyzanur Işbay Aydemir, Melike Dişsiz, Pınar Kumru

**Affiliations:** 1 Cemil Taşcıoğlu City Hospital – Istanbul, Türkiye.; 2 Istanbul Aydın University, Faculty of Health Sciences, Department of Nursing – Istanbul, Türkiye.; 3 University of Health Sciences, Hamidiye Faculty of Nursing, Department of Nursing – Istanbul, Türkiye.; 4 University of Health Sciences, Zeynep Kamil Women and Children Diseases Training and Research Hospital – İstanbul, Türkiye.

**Keywords:** Pregnancy, Endocrine disruptors, Attitude

## Abstract

**OBJECTIVE:**

The aim of this study was to determine the knowledge and attitudes of pregnant women about endocrine disruptors.

**METHODS:**

This cross-sectional and descriptive study was conducted with 313 pregnant women who visited the obstetrics clinic of a state hospital. Data were collected through face-to-face interviews using a Personal Information Form and the Endocrine Disruptors Attitude Scale. Data analysis was performed using SPSS 25.0, with a significance level of p<0.05.

**RESULTS:**

The average age of the pregnant women was 28.52±4.52 years. It was found that 62% of the pregnant women had more than 8 years of education, only 20.8% had prior knowledge about endocrine disruptors, and 30.7% of these obtained more information from social and visual media. Pregnant women scored an average of 76.26±9.33 on the Endocrine Disruptors Attitude Scale and were found to have a moderate protective attitude toward endocrine disruptors. It was determined that women who did not consume alcohol during pregnancy and were previously knowledgeable about endocrine disruptors scored significantly higher on the Endocrine Disruptors Attitude Scale (p<0.05).

**CONCLUSION:**

In this study, it was determined that the attitudes of pregnant women toward endocrine disruptors were generally at a moderate level, and that the level of knowledge about endocrine disruptors during pregnancy could have an effect on the attitude.

## INTRODUCTION

Endocrine disruptors (EDs) are chemicals that interfere with the endocrine system and can cause adverse health effects in healthy organisms or their offspring^1,2^. According to the World Health Organization (WHO), these substances can disrupt hormone regulation and enter the human body through various everyday sources such as plastics, cosmetics, pesticides, and food additives^3^. Increasing evidence links ED exposure during pregnancy to serious health risks, including gestational diabetes, pre-eclampsia, miscarriage, and low birth weight^4,5^. Given the sensitivity of the fetal developmental period, reducing exposure to these harmful agents during pregnancy is especially critical^5^.

Pregnant women represent a particularly vulnerable population due to both physiological changes and the potential long-term health consequences for the fetus. Despite the growing global awareness of ED-related risks, studies indicate that many pregnant women lack sufficient knowledge about EDs and how to prevent exposure^6^. For instance, a study in France found that while 74.2% of pregnant women acknowledged the importance of information about EDs, the majority reported feeling uninformed^7^.

Although EDs are linked to various environmental and global health concerns—impacting Sustainable Development Goals (SDGs) such as health and well-being (SDG 3), clean water and sanitation (SDG 6), responsible consumption and production (SDG 12), and climate action (SDG 13)^6^, this study focuses primarily on pregnancy-related risks. While SDGs provide a broader context, a detailed discussion of their relevance will be addressed in the discussion section.

In Turkey, research on pregnant women's knowledge and attitudes regarding EDs are quite limited. The existing literature generally focuses on general environmental health issues and falls short of providing information specific to a special and sensitive period such as pregnancy. This situation makes it difficult to develop targeted public health and women's health strategies and effective education programs for pregnant women in Turkey^8^.

This study aims to determine the knowledge and attitude levels of pregnant women in Turkey regarding EDs and, in this context, seeks to address the existing knowledge gap and establish a scientific foundation for health policies.

## METHODS

### Type of study

This cross-sectional and descriptive study was conducted to determine the knowledge and attitudes of pregnant women about EDs. This study followed the Strengthening the Reporting of Observational Studies in Epidemiology (STROBE) guidelines.

### Population and sample

The study population consisted of 9,477 pregnant women who visited the antenatal clinic of a public hospital affiliated with the Ministry of Health between November 1, 2023, and February 1, 2024. The sample consisted of 313 pregnant women aged 18 years and older who could speak and understand Turkish, were literate, had no communication barriers, and voluntarily agreed to participate in the study. The sample size (n=9,477) was calculated using a known sampling method with a 95%CI and a 5% margin of error.

### Data collection tools

Data were obtained through face-to-face interviews conducted in a separate room within the clinic with women who visited the maternity clinic of a state hospital affiliated with the Ministry of Health, where the study was conducted, for examination and follow-up. In this study, data were collected using a "Personal Information Form" and the "Endocrine Disruptors Attitude Scale (EDAS)."

In this study, a preliminary pilot study was conducted with five pregnant women to determine the comprehensibility of the EDAS and the Personal Information Form used prior to the main study, and these women were not included in the main study.

Personal information form: This form, consisting of 28 questions compiled by researchers through a review of the relevant literature, covers participants’ sociodemographic characteristics (age, education level, employment status, income level, smoking and alcohol use, regular medication use, etc.), obstetric characteristics (number of pregnancies and births, number of live births, number of miscarriages, current pregnancy status, etc.), and pregnant women's awareness of EDs (whether they know about EDs, sources of information about EDs, etc.)^3,4,8^.

Endocrine Disruptors Attitude Scale (EDAS): Developed by Miral et al., EDAS assesses adults’ attitudes toward EDs. The scale consists of two subscales, Consumer Behaviour and Nutrition and Hygiene, and 21 items. The "Consumer Behaviour" subscale consists of 11 items (1, 2, 7, 8, 9, 10, 11, 12, 13, 18, and 21), while the "Nutrition and Hygiene" subscale consists of a total of 10 items (3, 4, 5, 6, 14, 15, 16, 17, 19, and 20). There are no reverse-coded items in the scale. The scale has no cutoff value. The highest possible score on the 5-point (5=Strongly agree, 4=Agree, 3=Undecided, 2=Disagree, 1=Strongly disagree) Likert scale is 105, while the lowest possible score is 21. Higher scores indicate a positive attitude toward protecting oneself from EDs^9^. The Cronbach alpha coefficient for the internal consistency of the scale was reported as 0.85 in the original study and was calculated as 0.87 in this study.

### Statistical analysis

The study data were analyzed using Statistical Package for the Social Sciences (SPSS) version 25.0 software. Descriptive statistics (number, percentage, mean, and standard deviation) were used to examine individual and pregnancy-related characteristics. Student's t-test and one-way analysis of variance (ANOVA) test were applied to compare scale scores based on independent variables. The homogeneity in these measurements was assessed using the Levene test and the ANOVA-homogeneity test. A p<0.05 was considered statistically significant.

### Ethical considerations

Ethics committee approval (11.10.2023/138) and institutional work permit (no: 18.10.2023/251138364) were obtained from Zeynep Kâmil Women's and Children's Diseases Training and Research Hospital. The pregnant women were informed about the study in accordance with the Declaration of Helsinki, and their written and verbal consent was obtained.

## RESULTS

The mean age of the pregnant women participating in this study was found to be 28.52±4.52 years (min–max: 18–41). The majority of the participants (62%) had more than 8 years of education and were living in nuclear families (89.8%). More than half of the pregnant women had income equal to their expenses (58.5%) and were working in a paid job (64.2%). When evaluated in terms of obstetric history and health behaviors, it was determined that 13.4% of the participants had at least one miscarriage and 7.7% had a history of premature birth. It was determined that very few of the pregnant women smoked (9.3%) and consumed alcohol (1.9%) during pregnancy, while 39% used medication during pregnancy (Table 1).

**Table 1:** Individual and obstetric characteristics of the participants (n=313).

Age		Number (n)	Percentage (%)
Less than 28 years		129	41.2
28 years and older		184	58.8
Education level			
	8 years and less	119	38.0
	Above 8 years	194	62.0
Family type			
	Nuclear family	281	89.8
	Extended family	32	10.2
Economic situation			
	Income less than expenditure	60	19.2
	Income equal to expenditure	189	58.5
	Income more than expenditure	70	22.4
Employment status			
	Working	201	64.2
	Not working	112	35.8
Gestational age			
	20 weeks and under	164	52.4
	Over 20 weeks	149	47.6
Number of pregnancies			
	One pregnancy	202	64.5
	Two or more pregnancies	111	35.5
Miscarriage status			
	Yes	42	13.4
	No	271	86.6
Stillbirth status			
	Yes	3	1.0
	No	310	99.0
Preterm labor			
	Yes	24	7.7
	No	289	92.3
Smoking status during pregnancy			
	Yes	29	9.3
	No	284	98.1
Alcohol use during pregnancy			
	Yes	6	1.9
	No	307	98.1
Medicine use during pregnancy			
	Yes	122	39.0
	No	191	61.0
		**Mean**±**SD (min–max)**	
Gestational week		20.12±8.52 (8–40)	
Number of pregnancies		1.57±0.91 (1–6)	
Number of births		0.59±0.10 (0–6)	

SD: standard deviation.

When the participants’ awareness and attitudes on EDs were examined, 15.7% stated that they had received training on topics such as breastfeeding and baby care, while only 20.8% had prior knowledge about EDs. Among those who stated that they were aware, 30.7% stated that they mostly obtained this information through mass media such as social media and television (Table 2). In this study, the mean score of the pregnant women on the EDAS was 76.26±9.33 (range: 21–105), and it was determined that they had a moderately protective attitude toward EDs.

**Table 2:** Distribution of characteristics of participants regarding endocrine disruptors (n=313).

Characteristics		N	%
Status of receiving education during pregnancy			
Breastfeeding education		23	7.3
Birth preparation education		26	8.3
No		264	84.3
Status of knowledge about endocrine disruptors			
	Yes	65	20.8
	No	248	79.2
Level of knowledge about endocrine disruptors			
	Good	33	50.8
	Moderate	28	43.1
	Poor	4	6.2
Source of information about endocrine disruptors			
	Health professionals	47	15.0
	Social and visual media	96	30.7
	Internet	79	25.2
**Endocrine Disruptors Attitudes Scale and subdimensions**		**Mean**±**SD**	**Min–max**
Consumer behaviour subdimension		39.23±6.52	11–55
Nutrition and hygiene subdimension		37.03±5.17	10–50
Endocrine Disruptors Attitude Scale (EDAS)		76.26±9.33	21–105

SD: standard deviation.

Comparative analysis showed that there was no statistically significant difference in EDAS scores based on most sociodemographic or obstetric variables (p>0.05), except for alcohol use and prior knowledge about EDs. Participants who did not consume alcohol during pregnancy and those who had prior knowledge of EDs scored significantly higher on the EDAS, reflecting more protective attitudes (Table 3).

**Table 3:** Distribution of mean Endocrine Disruptors Attitude Scale scores by variables related to individual and obstetric characteristics.

Variables			Mean±SD	Test value/p
Individual characteristics EDAS				
Age		Less than 28 years (n=129)	75.51±10.07	t=-1.151
		28 years and above (n=182)	76.78±8.77	p=0.251
Education level		8 years and less (n=119)	75.83±9.37	t=-0.637
		Over 8 years (n=194)	76.52±9.32	p=0.524
Economic situation		Income less than expenditure (n=60)	76.15±7.69	
		Income equal to expenditure (n=183)	75.61±10.17	F=1.721
		Income more than expenditure (n=70)	78.04±8.15	p=0.181
Employment status		Working (n=201)	75.78±9.84	t=-1.121
		Not working (n=112)	77.12±8.31	p=0.223
Family type		Nuclear family (n=281)	76.58±9.17	t=1.812
		Extended family (n=32)	73.43±10.38	p=0.071
Obstetrics characteristics				
	Gestational age	20 weeks and under (n=164)	77.04±8.38	t=1.540
		Over 20 weeks (n=149)	75.44±10.24	p=0.125
	Number of pregnancies	One pregnancy (n=202)	75.83±9.21	t=-1.087
		Two or more pregnancies (n=111)	77.03±9.56	p=-0.278
	Miscarriage status	Yes (n=42)	76.49±9.46	t=0.142
		No (n=271)	76.23±9.33	p=0.887
	Preterm labor	Yes (n=24)	74.66±9.65	t=-0.871
		No (n=289)	76.39±9.31	p=0.385
	Smoking status during pregnancy	Yes (n=29)	75.71±8.68	t=-0.325
		No (n=284)	76.31±9.41	p=0.745
	Alcohol use during pregnancy	Yes (n=6)	69.33±6.83	**t=-2.618**
		No (n=307)	76.39±9.33	**p=0.050**
	Medicine use during pregnancy	Yes (n=122)	76.23±9.76	t=-0.037
		No (n=191)	76.27±9.08	p=0.971
	Status of knowledge about endocrine disruptors	Yes (n=65)	79.89±9.42	**t=3.587**
		No (n=248)	75.31±9.09	**p=0.000**

SD: standard deviation; Min: minimum; Max: maximum; EDAS: Endocrine Disruptors Attitude Scale; t: Student's t-test; F: One-way ANOVA; statistically significant values are denoted in bold.

## DISCUSSION

Exposure of pregnant women to EDs is an international concern^10^. International organizations such as the WHO and the International Federation of Gynecology and Obstetrics (FIGO) acknowledge that prenatal exposure to environmental chemicals is associated with adverse obstetric outcomes^1,11^. However, a lack of awareness regarding EDs is widespread due to insufficient knowledge about the harmful health effects these substances can cause^11-13^. Therefore, providing accurate information on EDs is considered critical for protecting maternal and child health^14^.

Health professionals are emphasized as key providers of accurate information and effective guidance to prevent pregnant women's exposure to EDs^10,11,15^. Nevertheless, there are limited studies in the literature investigating pregnant women's knowledge and attitudes toward EDs. This study also concludes that pregnant women need information about EDs, and that knowledge and attitudes are important factors.

In our study, it was found that only 20.8% of pregnant women had knowledge about EDs, with 30.7% primarily obtaining this information from social and visual media. This finding aligns with the study by Rouillon et al. in France, where 51% of participants were pregnant women and most had poor knowledge about EDs and exposure sources. In that study, 54.3% of women were unaware of EDs, while those informed primarily cited social media as their source^16^. Similarly, another study found that 40.8% of pregnant and postpartum women had knowledge of EDs, mainly obtained through social and visual media^17^.

Although there are many information sources on EDs, women perceive the quality and accuracy of these sources differently. Ashley et al. found that although pregnant women frequently use social and visual media to obtain information, they do not highly trust the information from these sources^18^. Similarly, in our study, health professionals—although regarded as strong sources—were the least utilized information providers^16^.

In a Serbian study by Živančević et al., 66.8% of pregnant and postnatal women had heard of EDs, but only 42.41% of them knew the meaning, while 33.2% had never heard the term. Participants stressed the health risks of ED exposure and the importance of pre-pregnancy education^19^. Our study findings align with previous research, reinforcing the need to educate pregnant women about EDs.

Miral and Koç reported that awareness and attitudes toward EDs were higher among women with higher education and income levels, those who were employed, and primiparous women^20^. Likewise, although our study did not find significant differences in attitudes based on age, education, income, or family type, it can be stated that older pregnant women, those with higher education and income levels, and those living in nuclear families had more positive attitudes toward EDs. Moreover, women who abstained from alcohol during pregnancy and who had knowledge about EDs showed more positive attitudes.

In Miral and Koç's study, 99.7% of women had not received education about EDs, and the mean score of the EDAS was 73.26±8.51, with subscale scores for consumer behaviour at 30.02±6.61 and nutrition and hygiene at 43.24±3.50^20^. The EDAS and subscale scores in our study yielded results similar to the literature.

## CONCLUSION

This study found that pregnant women's attitudes toward EDs were generally moderate, and knowledge about EDs during pregnancy may influence these attitudes. Therefore, providing comprehensive information and awareness training for pregnant women is important for maternal and fetal health. Health professionals are advised to play an active role and include counseling on EDs in prenatal care. Enhancing pregnant women's knowledge of EDs is key to protecting them from environmental risks. Thus, integrating ED education into preventive health services for women of reproductive age is recommended.

### Strengths and limitations

This study has many strengths, including being one of the few studies evaluating the knowledge and attitudes of pregnant women regarding EDs in Turkey. However, it also has some limitations. The first limitation is that the study was conducted at a single center, which limits the generalizability of the findings to other regions or the entire population. Second, the study used a cross-sectional design, which limits the ability to draw conclusions about causality or changes over time. Another limitation is that the data were collected based on participants’ verbal statements, which limits the objectivity of the data obtained.

## Data Availability

The datasets generated and/or analyzed during the current study are available from the corresponding author upon reasonable request.
